# SARS-CoV2 infectivity is potentially modulated by host redox status

**DOI:** 10.1016/j.csbj.2020.11.016

**Published:** 2020-11-20

**Authors:** Jaswinder Singh, Rajinder S. Dhindsa, Vikram Misra, Baljit Singh

**Affiliations:** a Department of Plant Science, McGill University, Ste Anne de Bellevue, Quebec H9X 3V9, Canada; b Department of Biology, McGill University, Montreal, Quebec H3A 1B1, Canada; c Western College of Veterinary Medicine, University of Saskatchewan, Saskatoon, Saskatchewan S7N 5B4, Canada; d Faculty of Veterinary Medicine, University of Calgary, Calgary, Alberta T2N 1N4, Canada

**Keywords:** SARS-CoV, COVID-19, Redox, Disulphide, Spike protein, ACE2, Cysteines

## Abstract

The current coronavirus disease (COVID-19) outbreak caused by Severe Acute Respiratory Syndrome Coronavirus-2 (SARS-CoV2) has emerged as a threat to global social and economic systems. Disparity in the infection of SARS-CoV2 among host population and species is an established fact without any clear explanation. To initiate infection, viral S-protein binds to the Angiotensin-Converting Enzyme 2 (ACE2) receptor of the host cell. Our analysis of retrieved amino acid sequences deposited in data bases shows that S-proteins and ACE2 are rich in cysteine (Cys) residues, many of which are conserved in various SARS-related coronaviruses and participate in intra-molecular disulfide bonds. High-resolution protein structures of S-proteins and ACE2 receptors highlighted the probability that two of these disulfide bonds are potentially redox-active, facilitating the primal interaction between the receptor and the spike protein. Presence of redox-active disulfides in the interacting parts of S-protein, ACE2, and a ferredoxin-like fold domain in ACE2, strongly indicate the role of redox in COVID-19 pathogenesis and severity. Resistant animals lack a redox-active disulfide (Cys133-Cys141) in ACE2 sequences, further strengthening the redox hypothesis for infectivity. ACE2 is a known regulator of oxidative stress. Augmentation of cellular oxidation with aging and illness is the most likely explanation of increased vulnerability of the elderly and persons with underlying health conditions to COVID-19.

## 1 Introduction

Since Ivanovsky’s discovery of viruses in 1890, these submicroscopic particles have been identified in virtually every type of ecosystem, causing acute or chronic infections in bacteria, fungi, plants and animals, including humans [1]. Viruses are highly diverse with respect to shape, genetic composition and mode of transmission [2]. However, once inside a host cell, they co-opt its metabolic machinery for their replication and release, and they use various mechanisms to spread. Viruses that spread rapidly and cause severe disease and mortality elicit fear and anxiety, due to a lack of specific and effective therapeutics, stressing not only economies but also entire civilizations. Viral disease in humans and animals can disrupt global food supply chains and exacerbate concerns regarding food security.

The ongoing COVID-19 pandemic has already infected more than 30 million and killed over 1 million people worldwide. Some abattoirs have suspended operations due to SARS-CoV2-infected workers, disrupting food-supply chains and heightening fear. SARS-CoV2 that causes COVID-19 appears to be more virulent than SARS-CoV for the SARS epidemic in 2003. It is known that SARS-CoV2 can infect humans, cats, dogs and ferrets but not bovine and swine [3], [4]. COVID-19 hits the elderly and people with underlying conditions more severely than the young and healthy ones. Reasons for this differential viral infectivity and disease severity are unclear.

Coronaviruses belong to the Coronaviridae family, with a single-stranded RNA genome housed in an envelope protein capsid [5]. SARS-CoV2, the seventh coronavirus known to be pathogenic to humans [6], is similar to SARS-CoV and MERS-CoV and belongs to the genus Betacoronavirus. Human Betacoronaviruses have many similarities in respect to their mode of action; however, differences in their genome and phenotypic structure could influence their pathogenicity and behavior [7]. The surface spike proteins of SARS-CoV2 are trimeric [8]. A recent structural study [8] showed that ACE2 has a short intracellular part, a transmembrane helical part and an extracellular peptidase domain (PD) part. To initiate infection, two S-proteins bind to the membrane-anchored dimer of ACE2 receptor. More specifically, the receptor binding domain (RBD) of spike protein binds to the peptidase domain of the membrane-anchored angiotensin-converting enzyme 2 (ACE2) receptor of the host cell. Significantly, for the present study, there is a ferredoxin-like fold domain between the transmembrane helix and the PD [8]. Cellular redox state is a major indicator of cell health and is mainly modulated by redox-active sulfhydryl/disulfide exchange and the latter is frequently mediated by thioredoxin system [9]. This prompted us to examine the presence of Cys residues in S-protein, especially in its RBD region, as well as in the peptidase domain of the ACE2 receptor. This primal interaction is the main focus of our study.

## 2 Results and discussion

To explore the spike glycoprotein of SARS-related coronaviruses, we retrieved the amino acid sequences of 20 spike proteins from a diverse range of coronaviruses that all use the same host receptor and cause disease in various organisms. Based on clustal analysis [10], spike proteins have significant sequence homology, ranging from 72 to 97% (Table 1). High sequence conservation (86%) between certain SARS-CoV and SARS-CoV2 epitopes was recently reported [11]. As cysteine residues have vital roles in structural stabilization, catalytic activity and post-translational modification of proteins, we focused our attention on these residues and their locations in the sequences (Table 1). Most of the spike proteins contain 37–40 Cys residues, 36 of which are conserved in the spike proteins of various SARS-coronaviruses (Table 1).

**Table 1:** Amino acid sequence, cysteine residues and percent similarity in the surface spike (SP) protein of different SARS-related coronaviruses.

Coronavirus	Amino Acids	Cysteine Residues (SP)	Cysteine Residues in RBD	Similarity with SARS-CoV2 (%)	Accession No. (database)
SARS-CoV2	1273	40	9	100.0	P0DTC2 (UniProt)
Bat-RaTG13	1269	40	9	97.7	QHR63300.2 (NCBI)
Bat-SARS-like	1255	40	9	78.1	ATO98157.1 (NCBI)
Bat- Rs3367	1256	39	9	77.9	U5WHZ7 (UniProt)
SARS-WIVI	1256	39	9	77.9	U5WI05 (UniProt)
Bat- HKU3	1242	37	7	77.9	Q3LZX1 (UniProt)
Bat-RsSHC014	1256	39	9	77.8	U5WLK5 (UniProt)
Bat-Rp3	1241	37	7	77.1	Q3I5J5 (UniProt)
Human-GZ02	1255	39	9	77.1	Q6TPE8 (UniProt)
SARS-CoV	1255	39	9	76.9	P59594 (UniProt)
SARS-Urbani	1255	39	9	76.9	A0A3G5BJ39 (UniProt)
SARS-MA15	1255	39	9	76.9	D2E235 (UniProt)
SARS-Civet	1255	39	9	76.8	Q3ZTE0 (UniProt)
Bat-Rmi	1241	37	7	76.7	Q0QDX9 (UniProt)
Bat-279	1241	37	7	76.7	Q0Q475 (UniProt)
SARS-A022	1255	39	8	76.7	Q4JDM3 (UniProt)
SARS-Rs672	1238	38	7	76.2	D2DJW4 (UniProt)
Bat-Rf1	1238	37	7	75.7	Q0QDZ0 (UniProt)
Bat-CoV73	1238	37	7	75.5	Q0Q484 (UniProt)
Bat-bm48	1255	40	9	72.6	E0XIZ3 (UniProt)

**Fig. 1:**
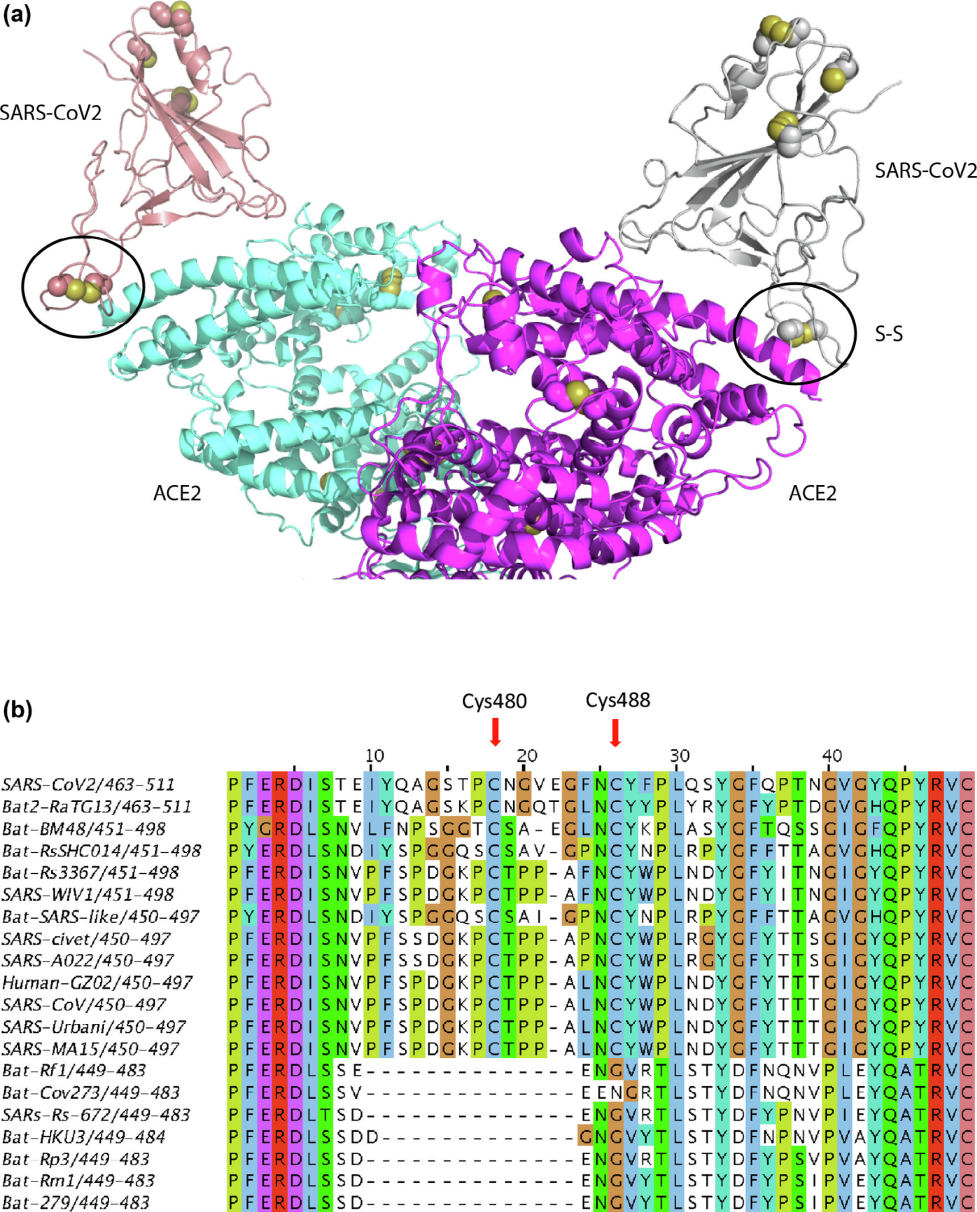
a: Ribbon representation of the complex of the receptor binding domain (RBD) of the surface spike glycoprotein (S-protein) of SARS-CoV2 bound to the extracellular domain of the human ACE2 receptor (PDB code: 6m17; Ref. [8]). The side-chain of cysteine amino acids are shown in spacefill. In the S-protein, one disulfide (formed by Cys480 and Cys488) is in a region that interacts with ACE2 protein; this disulfide is conserved in the S-proteins of the SARS coronavirus. Mutation of any of the Cys may influence formation of the protein complex and thus infection. b: Alignment of Receptor Binding Domain (RBD) of surface spike protein from SARS-related coronaviruses. Cysteine residues with redox potential are denoted with arrows.

The RBD region of spike proteins spans amino acids 319 to 541, with eight cysteine residues, six of which form three disulfide bridges (Cys336-Cys361, Cys379-Cys432, Cys391-Cys525) that seemed to have a structural role. The other two Cys residues, Cys480 and Cys488, were not visible in the electron density map, except Cys488 in one of the monomers (Fig. 1a), implying regions were conformationally flexible in structure. Analysis of several structures of the RBD region complexed with the ACE2 receptor [8], [12] revealed that Cys480-Cys488 pair had an important role in the loop region that directly participates in binding to the host receptor [12]. Computational modeling and biophysical measurements recently suggested that the SARS-CoV2 RBD has higher binding affinity to the receptor, ACE2, than that of SARS-CoV [7]. This may be related to the difference in the number of amino acids present between Cys480 and Cys488 and the length of the loop formed by the disulfide bridge formed by these two cysteines. For example, SARS-CoV RBD lacks one residue in the region between Cys480 and Cys488 and this would be expected to alter the configuration of the loop formed by the Cys480-Cys488 disulfide bond which in turn could influence the ability of the spike protein to engage the receptor and thus contribute to differences among species in viral infectivity and proliferation. Considerable differences in RBD sequences and binding affinity of various coronaviruses have been attributed to the viral tropism [7].

We further analyzed this variable region of the RBD and identified a deletion of 12–14 amino acids, including one cysteine, Cys480, that was exclusively lost in the spike proteins of eight coronaviruses. The other cysteine, Cys488, was substituted by glycine (Fig. 1b). Another important variation included the difference in the number of amino acids between Cys480 and Cys488 (Fig. 1b). Except SARS-CoV2 and Bat2-RaTG13 protein sequences, all other protein sequences that do not contain the deletion have one less amino acid residue between Cys480 and Cys488. These RBD variations could alter the functional properties of the spike proteins.

Receptor recognition is the first step of viral infection and one of the most important determinants of transmission ability, either human-to-human or cross-species. Therefore, any changes in this region would influence viral infection and transmission, as recently predicted [13]. Considerable differences in RBD sequences and binding affinity of various coronaviruses have been attributed to the viral tropism [7], [14]. Perhaps the disulfide bond between Cys480 and Cys488 has a crucial role in inducing a conformational change in the loop to favor interaction with the receptor. This disulfide bond is certainly located in a strategic position that could be regulated by cellular redox systems.

**Fig. 2:**
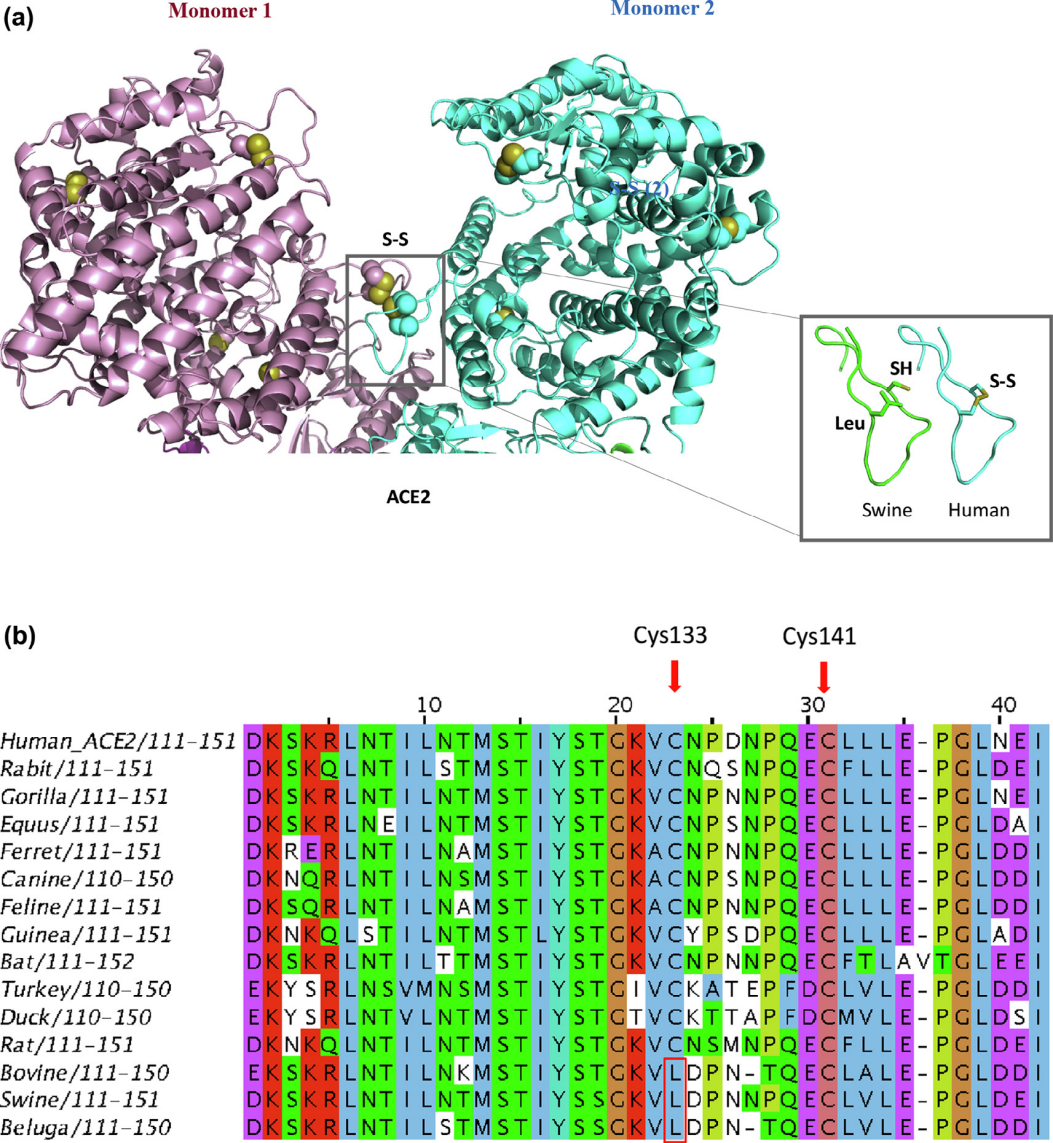
a: Ribbon representation of the extracellular domain of the homodimeric human ACE2 receptor (PDB code: 6m17; Ref. [8]). Sidechain of cysteine amino acids are shown in spacefill. The ACE2 protein has three disulfides. One disulfide participates in the interaction between monomers. A model of the loop with the mutated amino acid is shown on the right of the figure. The absence of disulfide may influence the conformational stability of the loop, with implications for dimer formation. b: Alignment of host receptor Angiotensin Converting Enzyme II (ACE2) sequences from different organisms. Cysteine residue (Cys133) replaced with leucine in cattle, swine and beluga is shown in the red box. Disulfide between Cys133 and Cys141 has been predicted for redox potential and denoted by red arrows. (For interpretation of the references to colour in this figure legend, the reader is referred to the web version of this article.)

We also aligned amino acid sequences of ACE2 receptors from 16 vertebrate coronavirus host species; the sequence of the human ACE2 receptor had 65 to 99% similarity with other species, with gorilla being the closest (Table 2). Notably, the human ACE2 receptor sequence has eight cysteine residues, of which six are conserved across ACE2 receptors of other species (Fig. 2b) and are involved in the formation of three disulfide bonds [11], [15]. One of these disulfides (Cys133–Cys141) forms part of a loop (Fig. 2a) at the dimer interface [8]*.* We noted sequence diversity between Cys133 and Cys141 among ACE2 of various organisms. Both cysteine residues were conserved, except in pigs, belugas and cattle where Cys133 has been substituted by a leucine (Table 2, Fig. 2b). Interestingly, cattle and swine are impervious to SARS-CoV2 infection, whereas dogs, cats and ferrets are susceptible [3], [4]. Thus, the ability to make Cys133-Cys141 disulfide bond aligns with susceptibility to COVID-19. The strategic position of the disulfide in the ACE2 structure and its absence in organisms not susceptible to SARS-CoV2, implies it has regulatory potential via redox systems (Fig. 2a). Thus, the differential ability of the viral spike protein to engage ACE2 receptor may underlie differences in the susceptibility of various animal species to SARS-CoV2 infection, as well as the ability of SARS-related coronaviruses to jump from animals to humans [4], [16]*.* The conserved cysteine residues in the receptor and the virus spike protein may determine SARS-CoV2 activity during binding and activation of host receptors, as well as viral transmission. The role of cysteine residues could be regulated through changes in the redox status of the cellular environment.

**Table 2:** Amino acid sequence, cysteine residues and percent similarity in the host receptor Angiotensin Converting Enzyme II (ACE2) sequences.

Receptor Host	Amino Acids	No. of Cysteine Residues	Similarity with Human_ACE2 (%)	UniProt Accession No.
Human	805	8	100	Q9BYF1
Gorilla	805	8	99	G3QWX4
Equus	805	9	89	F6V9L3
Feline	805	8	85	Q56H28
Rabbit	805	8	85	G1TEF4
Canine	804	8	84	J9P7Y2
Ferret	805	8	83	Q2WG88
Rat	805	9	82	Q5EGZ1
Swine	805	8	82	K7GLM4|
Beluga	804	8	81	A0A2Y9M9H3
Bovine	804	8	81	Q58DD0
Bat	807	7	81	G1PXH7
Guinea	794	8	76	H0VSF6
Duck	653	10	67	U3J4G2
Turkey	807	10	65	G1NPB8

The involvement of cysteines in redox activity through reversible disulfide bond formation and many other types of oxidation such as sulfenic acid formation has been studied rigorously in plant [17]*,* animal and microbial systems [18]. Several extracellular antioxidants, including paraoxonase (PON), thioredoxin (Trx), Trx reductase (TrxR), glutaredoxin (Grx), extracellular superoxide dismutase (EC-SOD), and extracellular glutathione (GSH), eliminate reactive oxygen species (ROS) [19], [20]. Cellular disulfides are mainly reduced by the thioredoxin (Trx) system. Thioredoxin is a low molecular-weight disulfide protein that undergoes sulfhydryl/disulfide exchange reactions with target proteins. Consequently, Trx has a regulatory role in a variety of processes, ranging from general metabolism and antioxidant metabolism to cell signaling [21]. Among other activities, Trx regulates photosynthesis [22], mitigates allergenicity [23], seed germination and improves the quality of bread and beer [24]. There is evidence for a role for Trx in human diseases and increased expression of Trx suppresses Parkinson’s disease in mice and has been proposed as a treatment [25]. Associations of ROS and influenza virus replication has been documented and ROS inhibitors have been proposed for suppression of influenza A virus-induced lung disease [26]. Oxidative stress has been implicated in several major age-related conditions, including those involving cardiovascular, pulmonary, kidney disease and nerve tissues [9].

**Fig. 3:**
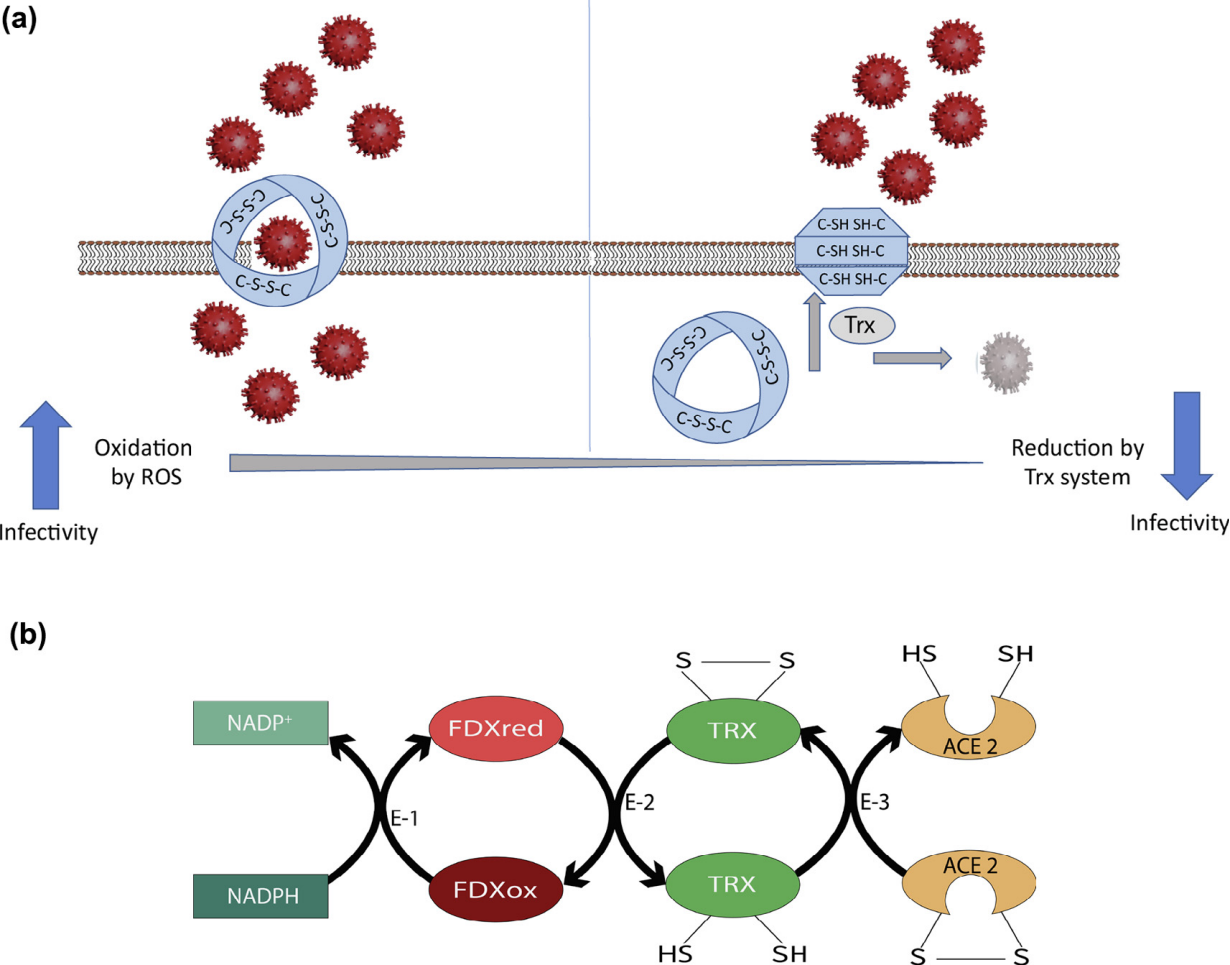
a: Proposed Trx-dependent redox model for interaction of SARS-CoV2 with cell membrane receptor ACE2; Left panel showing the successful entry of SARS-CoV2 into the cell due to appropriate protein folding and active ACE2 receptor with intact disulfide bonds in an oxidative cellular environment; Right panel showing restricted entry of SARS-CoV2 due to inactive ACE2 receptor incorrect protein folding as disulfide bridges reduced to sulfhydryl bonds by the action of the Trx system. Escaped SARS-CoV2 particles are shown as disintegrated virus molecules, due to the action of the Trx system. b: Suggested path of electron flow during thioredoxin-linked reduction of ACE2. ACE2 receives electrons from reduced thioredoxin (TRX). Oxidized TRX receives electrons from reduced ferredoxin (FDX). Finally, oxidized ferredoxin receives electrons from NADPH. E-1, NADPH-linked ferredoxin reductase; E-2, ferredoxin-linked thioredoxin reductase; E-3, thioredoxin-linked ACE2 reductase.

Cysteine-rich spike glycoproteins of SARS-CoV2 and its host receptors are likely to be influenced by redox-active disulfides in a Trx-associated fashion, or by other redox systems mentioned above. The Trx system protects against oxidative stress by providing electrons to thiol-dependent enzymes that remove ROS [9]. Therefore, SARS-CoV2 infection and the associated disease follow a similar redox-associated path. If so, this could explain associations among age, COVID-19 and mortality. A role for thioredoxin in SARS-CoV2 biology and COVID19 severity is strengthened by two observations. First, deficiency of ACE2 expression in mice results in high levels of oxidation stress [27]. Second, the transmembrane part of ACE2 contains a ferredoxin-like fold domain [8]. Ferredoxin is known to pass electrons to thioredoxin which, in turn, reduces disulfide to sulfhydryls [21]. Based on our observation regarding disulfides in spike protein (Fig. 1a) and ACE2 receptor (Fig. 2a), a Trx-dependent redox model could be created (Fig. 3a). The path of electron flow during Trx-mediated disulfide reduction is shown in Fig. 3b.

In one scenario, the host receptor (ACE2) with three disulphide bonds through conserved cysteines could require an oxidative environment to keep its disulfide bonds intact and hence allow SARS-CoV2 to infiltrate target cells. Consequently, higher ROS concentrations in the elderly or those with underlying health conditions would predispose them to more vigorous SARS-CoV2 infection, replication and disease. Higher ROS concentrations could also yield an appropriate environment for SARS-CoV2 surface spike protein to be operational, especially if its disulfide bonds are involved in redox. Taken together, an ROS-rich cellular environment would promote SARS-CoV2 infections and more lethal COVID-19. In contrast, a reducing or antioxidant environment may minimize the receptors’ interaction with SARS-CoV2. This reducing environment would also cause reduction of SARS-CoV2′s own disulfide bonds, leading to its disintegration. The Trx system has been documented to accomplish redox adjustments in such circumstances, as manifested in many similar situations and reviewed by Buchanan [18]. It remains to be experimentally verified whether redox status of the host indeed plays a role in SARS-CoV2 biology and COVID-19 infection. Cysteine-rich surface spike glycoproteins of SARS-CoV2 and its host receptors are likely to be influenced by redox-active disulfides in a Trx-associated fashion, or by other redox systems mentioned above. Thus, increased oxidation associated with aging, smoking and various co-morbidities may contribute to higher susceptibility to COVID-19. Potential Trx-dependent redox changes accompanying viral replication and infection warrant attention, as they may yield insights into the mechanism of action of the virus and appropriate therapeutic regimens. Antioxidant therapy could decrease disease severity by interfering with entry of the virus into host cells, a crucial first step in the establishment of infection.

A recently published report summarized various genetic strategies including associations between markers, blood groups and gene variants (*IFNAR2*, *OAC*, *TYK2* and *SLC6Z20*) with COVID-19 cases to uncover the underlying causes of disparity in the infectivity of SARS-CoV2, but was unable to draw any definitive conclusions [28]. There is still much to learn regarding basic mechanisms responsible for age-related susceptibility and contagiousness of SARS-CoV2 to develop effective, evidence-based prophylaxis and treatments. In our opinion, a better understanding of the role of conserved cysteines and ROS crosstalk between target cell and SARS-CoV2 may yield molecular clues to interfere with this primal step. Furthermore, perhaps CRISPR-based technologies could be applied to target and modify specific cysteine sequences of RBD region of spike protein (Cys480-Cys488) and receptors (Cys 133-Cys141) of appropriate model animals to elucidate the underlying mechanisms. There may also be value in modulating the redox activity via a Trx system to improve human health by adoption of appropriate prevention and treatment therapies. For example, improving the expression of thioredoxin reductase may be a promising therapeutic target for the development of novel and effective treatment strategies, as noted for Parkinson's disease [29]. As mitochondria are a major redox organelle, genes implicated in mitochondrial function could be altered via the CRISPR/cas system. The latter approach could also be used to identify unknown genes and their protein products involved in COVID-19 progression and development, with potential to decrease, restore and improve gene expression, perhaps preventing or treating COVID-19.

## Funding

J. Singh acknowledges the financial support by a grant from National Sciences and Engineering Research Council of Canada Collaborative Research and Training Experience (CREATE)

## Competing interests

Authors declare no competing interests.

## 5 Data and materials availability

All data is available in the main text.

## CRediT authorship contribution statement

**Jaswinder Singh:** Conceptualization, Investigation. **Rajinder S. Dhindsa:** Conceptualization, Investigation. **Vikram Misra:** Investigation. **Baljit Singh:** Investigation.
